# Saturated fatty acids induce insulin resistance in podocytes through inhibition of IRS1 via activation of both IKKβ and mTORC1

**DOI:** 10.1038/s41598-020-78376-1

**Published:** 2020-12-10

**Authors:** Benoit Denhez, Marina Rousseau, Crysta Spino, David-Alexandre Dancosst, Marie-Ève Dumas, Andréanne Guay, Farah Lizotte, Pedro Geraldes

**Affiliations:** 1 From the Research Center of the Centre Hospitalier Universitaire de Sherbrooke, Université de Sherbrooke, 3001 12e Ave Nord, Sherbrooke, QC J1H 5N4 Canada; 2 Division of Endocrinology, Department of Medicine, Université de Sherbrooke, 3001 12e Ave Nord, Sherbrooke, QC J1H 5N4 Canada

**Keywords:** Mechanisms of disease, Insulin signalling, Podocytes, Diabetic nephropathy, Fatty acids

## Abstract

Diabetic nephropathy (DN), a microvascular complication of diabetes, is the leading cause of end-stage renal disease worldwide. Multiple studies have shown that podocyte dysfunction is a central event in the progression of the disease. Beside chronic hyperglycemia, dyslipidemia can induce insulin resistance and dysfunction in podocytes. However, the exact mechanisms of free fatty acid (FFA)-induced podocyte insulin unresponsiveness are poorly understood. We used a type 2 diabetic mouse model (*db/db*) and mouse podocytes exposed to palmitic acid for 24 h followed by an insulin stimulation. Renal function and pathology were evaluated at 25 weeks of age to confirm the DN development. Our results demonstrate that saturated FFA activated the serine/threonine kinases IκB kinase (IKK)β/IκBα and mTORC1/S6K1, but not protein kinase C and c-jun N-terminal kinase, in podocytes and glomeruli of *db/db* mice. Activation of both kinases promoted serine 307 phosphorylation of IRS1, a residue known to provoke IRS1 inhibition. Using IKK, mTORC1 and ceramide production inhibitors, we were able to blunt IRS1 serine 307 phosphorylation and restore insulin stimulation of Akt. In conclusion, our results indicate that FFA and diabetes contribute to insulin resistance through the activation of IKKβ and S6K1 leading to podocyte dysfunction and DN.

## Introduction

Diabetic nephropathy (DN) is the worldwide leading cause of end-stage renal disease^1^ and is viewed as a detrimental health problem. While chronic hyperglycemia is one of the main risk factors for developing DN^2^, multiple clinical trials showed that insulin resistance, a common characteristic of type 2 diabetes, is an independent risk factor in developing chronic kidney disease^3^. Dyslipidemia in diabetic patients is commonly characterized by elevated circulating levels of free fatty acids (FFA) and triglycerides (hypertriglyceridemia)^4^. The lipotoxicity due to elevated FFA levels is a major factor and a key contributor to insulin resistance including the kidney^5,6^.

Palmitic acid (C16:0) is the most abundant circulating saturated FFA in human plasma^7^. High concentrations of palmitate can induce insulin resistance through multiple mechanisms. Mainly, FFA and palmitate increase oxidative stress production, activate protein kinase C (PKC), nuclear factor-kappa B (NF-κB), c-Jun N-terminal kinase (JNK) and mammalian target of rapamycin (mTOR) pathways. These signaling pathways have been shown to enhance serine phosphorylation of the insulin receptor substrate-1 (IRS1) causing insulin action inhibition. In normal volunteers, lipid injections led to insulin resistance in skeletal muscle, which was associated with activation of PKC-θ and serine 1101 phosphorylation of IRS1^8^. JNK activation in the liver, muscle and adipose tissue of obese *ob/ob* mice is linked with insulin resistance and phosphorylation of serine 307 of IRS1, while deletion of JNK1 protected these mice from insulin resistance^9^. In a type 2 diabetes mouse model (*db/db*), mTOR complex 1 (mTORC1) and S6K1 were chronically activated in the liver which was associated with insulin resistance and serine 1101 phosphorylation of IRS-1. Moreover, genetic deletion of S6K1 in mice fed with a high fat diet prevented both insulin resistance and serine 1101 phosphorylation of IRS1^10^. In other insulin sensitive tissues, mTORC1 activation led to the phosphorylation and stabilization of growth factor receptor-bound protein 10 (Grb10) causing IRS1 serine phosphorylation and insulin action inhibition^11,12^. The IκB kinase (IKK) complex is the major multiplex core for NF-κB activation composed of two serine–threonine kinases (IKKα and IKKβ) and the regulatory subunit NEMO (also known as IKKγ). The IKK complex regulates the phosphorylation of various IκB and NF-κB proteins, amongst other substrates. In response to oxidative stress, IKKβ is an important regulator of NF-κB-mediated pro-apoptotic actions^13^. Mice fed with high fat diet exhibited activation of IKKβ and insulin resistance in the liver. Moreover, specific genetic deletion of IKKβ in hepatic cells prevented inflammation-induced insulin resistance^14^. IKKβ is also known to inhibit insulin action through reduction of phospho-tyrosine residues of IRS1^15^. However, how these palmitate-activated signaling pathways by lead to renal dysfunction are not fully understood.

The glomerular podocytes are highly specialized epithelial cells that are critical in maintaining the integrity of the glomerular filtration barrier. Reduced podocyte density in the glomerulus has been shown to be one of the strongest predictors of the progression of DN^16^. Multiple studies in animal models of DN showed that podocyte cell death is an early event of the disease. Using mice with podocyte-specific deletion of the insulin receptor, Welsh and collaborators clearly showed the importance of insulin signaling in podocyte function^17^. Moreover, we and other have shown that insulin action inhibition was associated with loss of podocytes in DN^18,19^. Interestingly, it has been reported that disruption of the glomerular filtration barrier strongly correlates with the increase in circulating palmitic acid^20^. In addition, increased expression of fatty-acid-binding proteins specifically in podocytes is associated with proteinuria in patients with obesity-related glomerulopathy and in a type 2 diabetic mouse model (*db/db*)^21^. Besides insulin resistance, others have shown that enhanced endoplasmic reticulum stress, protein phosphatase PP2A and mTOR lysosomal localization are potential mechanisms for podocyte cell death caused by palmitate exposure^22–25^. However, the mechanisms by which palmitate can lead to insulin resistance in these cells are not fully elucidated.

In this study, we hypothesized that FFA would increase serine phosphorylation of IRS1 in cultured podocytes and the kidney of a type 2 diabetic mouse model causing insulin resistance and renal dysfunction. Furthermore, our study intended to identify the molecular mechanisms underlying palmitate-induced insulin unresponsiveness in renal podocytes.

## Results

### Leptin receptor deficient mice exhibited renal dysfunction and pathology

**Figure 1:**
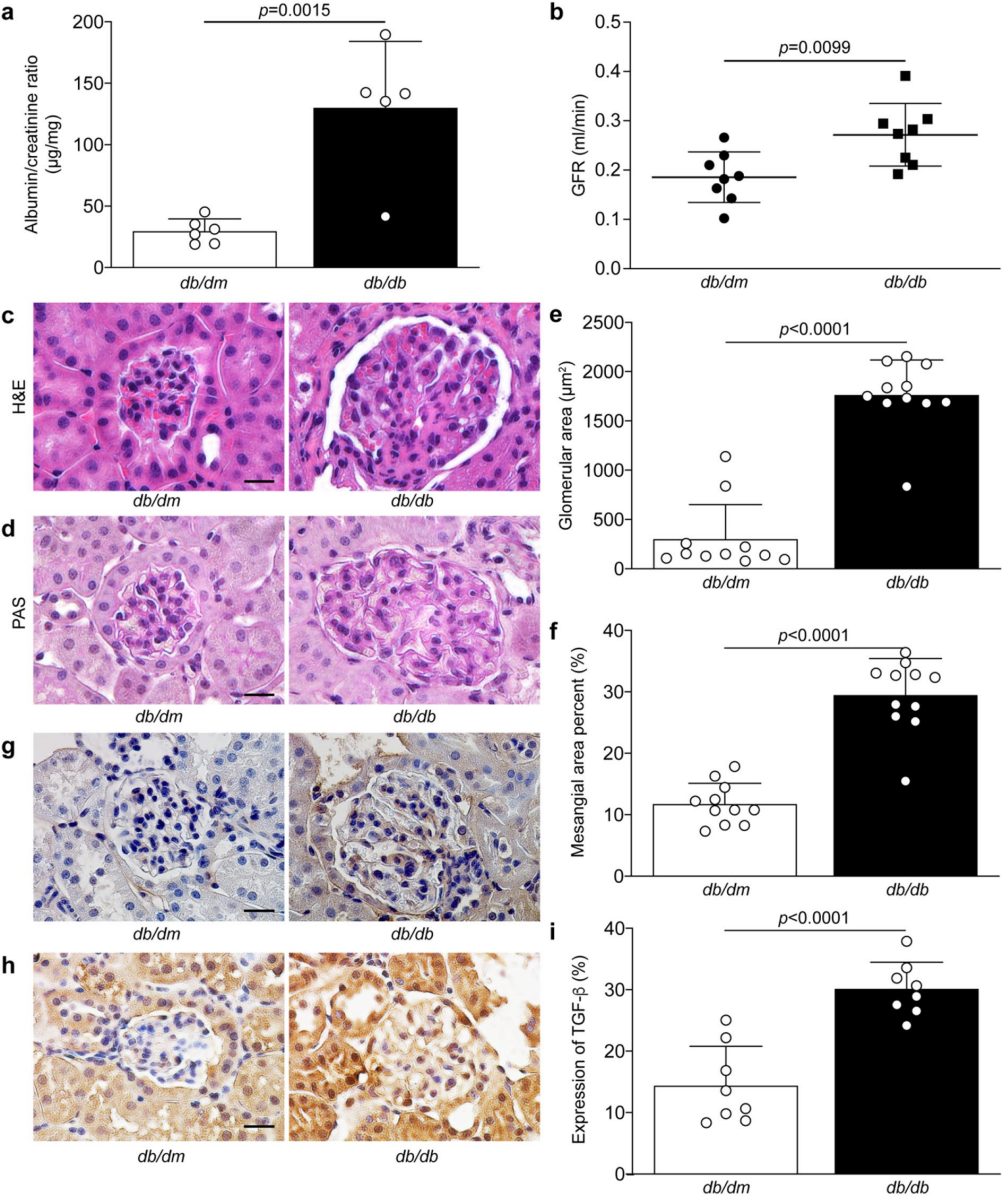
Renal function and glomerular pathology of nondiabetic *db/dm* and type 2 diabetic *db/db* mice. (**a**) Albumin/creatinine ratio and (**b**) glomerular filtration rate were performed to evaluate renal function. Renal cross-sections of 25 weeks of age *db/dm* and *db/db* mice were stained with (**c**) hematoxylin & eosin and (**d**) periodic acid-Schiff to measure (**e**) glomerular hypertrophy and (**f**) mesangial cell expansion. Immunohistochemistry using antibody against (**g**) collagen type IV (Col IV) and (**h**, **i**) TGF-β expression was quantified. Results are shown as mean ± SD of 5–6 (**a**), 8 (**b**, **h**, **i**), and 11 (**c**, **d**, **e**, **f**, **g**) mice per group. Scale bar = 10 μm.

In order to evaluate the effect of FFA exposure in the renal tissue, we have used a type 2 diabetic animal model, the leptin receptor deficient (*db*/*db*) mice, which is known to exhibit renal dysfunction and pathology^26^. Urinary albumin levels and glomerular filtration rate were used to evaluate both renal damage and renal function. As expected, we confirmed that at 25 weeks of age *db*/*db* mice exhibited higher levels of urinary albumin (Fig. 1a) and elevated glomerular filtration rate (Fig. 1b) as compared to *db*/*dm* littermate control mice. Besides renal dysfunction, *db*/*db* mice displayed glomerular hypertrophy (Fig. 1c,d), mesangial expansion (Fig. 1e,f), elevated collagen type IV (Fig. 1g) and TGF-β (Fig. 1h,i) expression in the glomeruli, all markers of renal pathology associated with diabetic nephropathy.

### Type 2 diabetes and podocyte exposure to FFA blunted insulin signaling and increased serine 307 phosphorylation of IRS1

**Figure 2:**
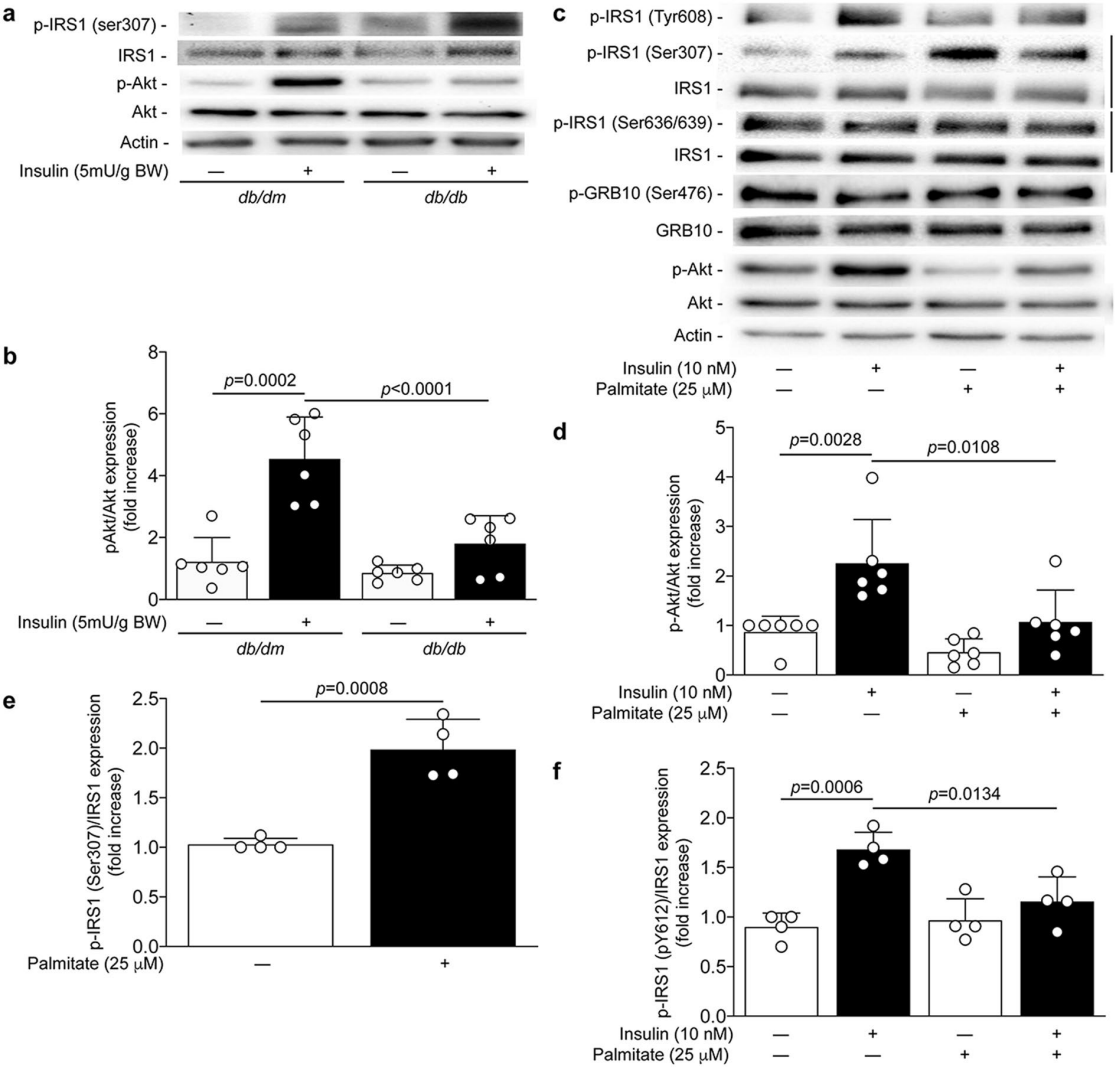
FFA and palmitate exposure induced insulin resistance. Expression of phospho-IRS1 (ser307), phospho-IRS1 (Tyr608), IRS1, phospho-Akt, Akt and actin were detected by (**a**, **c**) immunoblot and (**b**, **d**, **e**, **f**) densitometry quantitation was measured from (**a**–**b**) isolated glomeruli stimulated with insulin injection (I.V.) for 15 min in 25 weeks ols *db/dm* and *db/db* mice. at 25 weeks of age of nondiabetic *db/dm* and diabetic *db/db* mice as well as from (**c**, **d**, **e**, **f**) mouse podocytes exposed to palmitate for 24 h and then stimulated with insulin for 5 min. Results are shown as mean ± SD of 6 (**a**, **b**) mice per group and 4–6 (**c**, **d**, **e**, **f**) independent experiments.

To evaluate if the *db*/*db* mice are insulin resistant in the kidney, insulin (5 mU/g of BW) was injected systemically and the renal glomeruli were isolated after 15 min. We observed that the phosphorylation of Akt in the renal glomeruli was decreased in *db/db* mice compared to *db/dm* mice (Fig. 2,b). The reduced activity of Akt following insulin stimulation was associated with increased expression of serine 307 phosphorylation of the IRS1 (Fig. 2a), a residue phosphorylation known to be related to insulin resistance. In addition, podocytes are highly insulin-sensitive cells and insulin signaling actions are essential for their function. Podocytes exposed to a high dose of palmitate (750 µmol/L) has been shown to promote insulin resistance^20^. We have confirmed that treatment with 25 µmol/L of palmitate prevented insulin-induced Akt phosphorylation by 75% in cultured podocytes (*p* < 0.0001) (Fig. 2c,d). Inhibition of insulin actions was associated with a 98% increase of serine 307 phosphorylation of IRS-1 (*p* = 0.008) (Fig. 2c,e) and a complete inhibition of tyrosine 608 phosphorylation following insulin stimulation (*p* = 0.0257) (Fig. 2c,f), without affecting phosphorylation of Grb10 and serine 636/639 of IRS1 (Fig. 2c).

### Palmitate activated both mTORC1 and IKKβ pathways in podocytes

**Figure 3:**
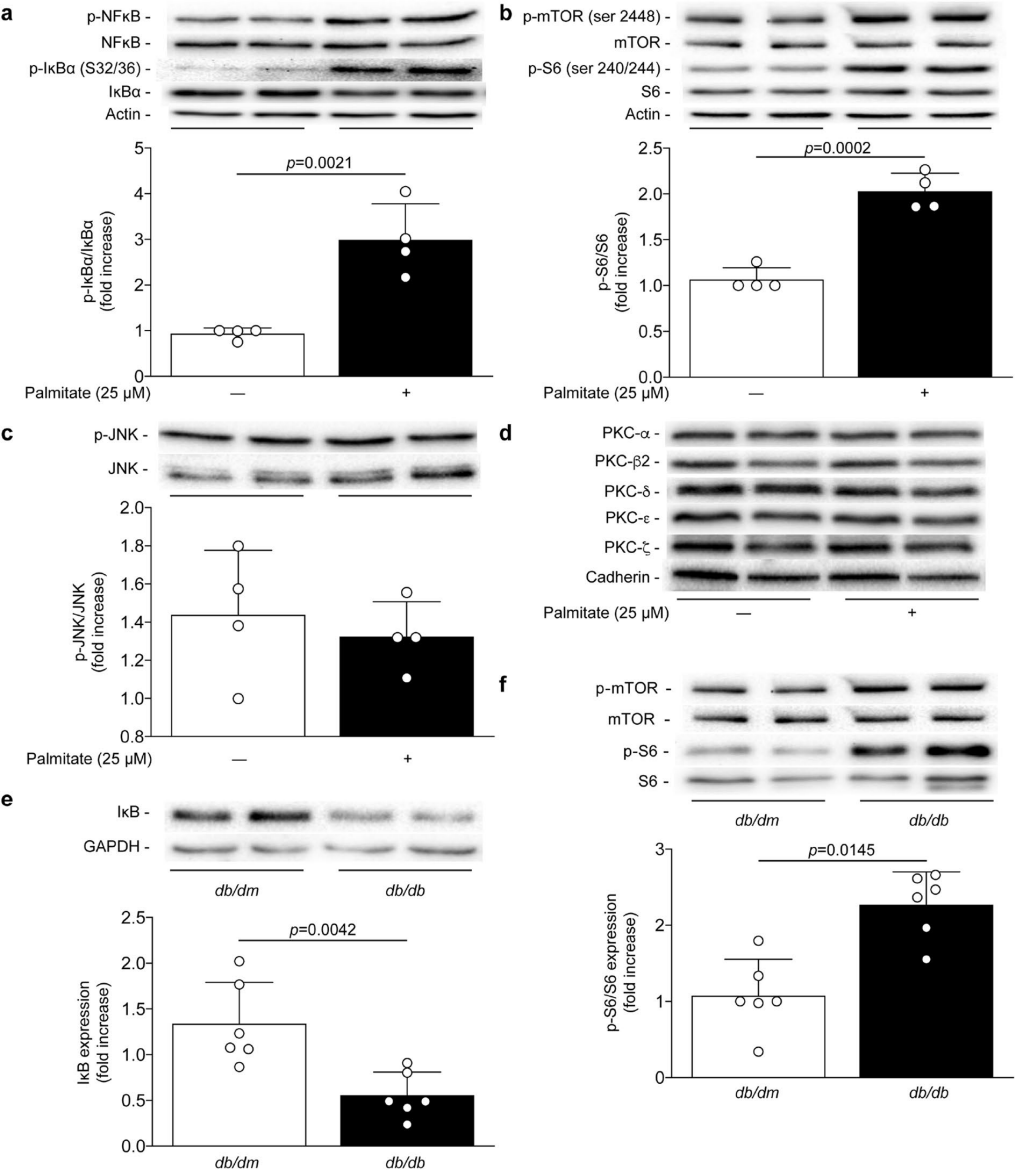
Activation of IκBα and mTORC1 caused by palmitate in podocytes and diabetic glomeruli. Expression of phospho-NF-κB, NF-κB, phospho-IκBα (ser32/36), IκBα, phospho-mTOR (ser2448), mTOR, phospho-S6 (ser240/244), S6, phospho-JNK, JNK, PKCα, PKCβ, PKCδ, PKCε, PKCζ, cadherin and actin were detected by immunoblot and densitometry quantitation was measured from (**a**, **b**, **c**, **d**) mouse podocytes exposed to palmitate for 24 h as well as from (**e**, **f**) isolated glomeruli at 25 weeks of age of nondiabetic *db/dm* and diabetic *db/db* mice. Results are shown as mean ± SD of 4 (**a**, **b**, **c**, **d**) independent experience and 6 (**e**, **f**) mice per group.

Multiple serine/threonine kinases have been shown to directly phosphorylate IRS1. We verified the effect of palmitate exposure on the activation of IKK mTORC1, PKC and JNK. Treatment with palmitate significantly increased IκBα serine 32/36 phosphorylation by threefold (*p* = 0.0021), which is believed to be a specific marker of IKKβ activation^27^ (Fig. 3a). One downstream target of IKK activity is the activation of the NF-κB pathway. Therefore, increased phosphorylation of IκBα was associated with enhanced phosphorylation of serine 536 of NF-κB by 91% following palmitate exposure (*p* = 0.0012; Fig. 3a). Treatment of podocytes with palmitate also significantly increased the phosphorylation of serine 2448 of mTOR by 74% (*p* = 0.0082; Fig. 3b) and subsequently the S6 ribosomal protein serine 240/244 phosphorylation by 90% (*p* = 0.0002; Fig. 3b). These data suggest that palmitate can activate the mTORC1 complex signaling pathway. Interestingly, palmitate exposure had no effect on the phosphorylation of JNK in podocytes (Fig. 3c) nor did it cause membrane translocation (as a marker of activation) of various PKC isoforms known to inhibit insulin signaling (Fig. 3d). In order to corroborate our cell culture findings in vivo, renal glomeruli of type 2 diabetic mice were isolated and immunoblot was performed. Due to limitation of a good specific antibody in vivo for phosphorylation of IκBα, we measured the expression of IκB. Our data demonstrated that the expression of IκB is decreased by 58% (*p* = 0.0042) in renal glomeruli of *db/db* mice compared to control littermates (Fig. 3e). These data suggest that IκB is degraded, therefore releasing its association with NF-κB. Moreover, renal tissue of our type 2 diabetic mouse model exhibited elevated levels of mTOR and S6 phosphorylation by 1.5-fold (*p* = 0.0145; Fig. 3f).

### Inhibition of IKK/IκBα activity prevented palmitate-induced serine 307 phosphorylation of IRS1 and partially restored insulin signaling actions

**Figure 4:**
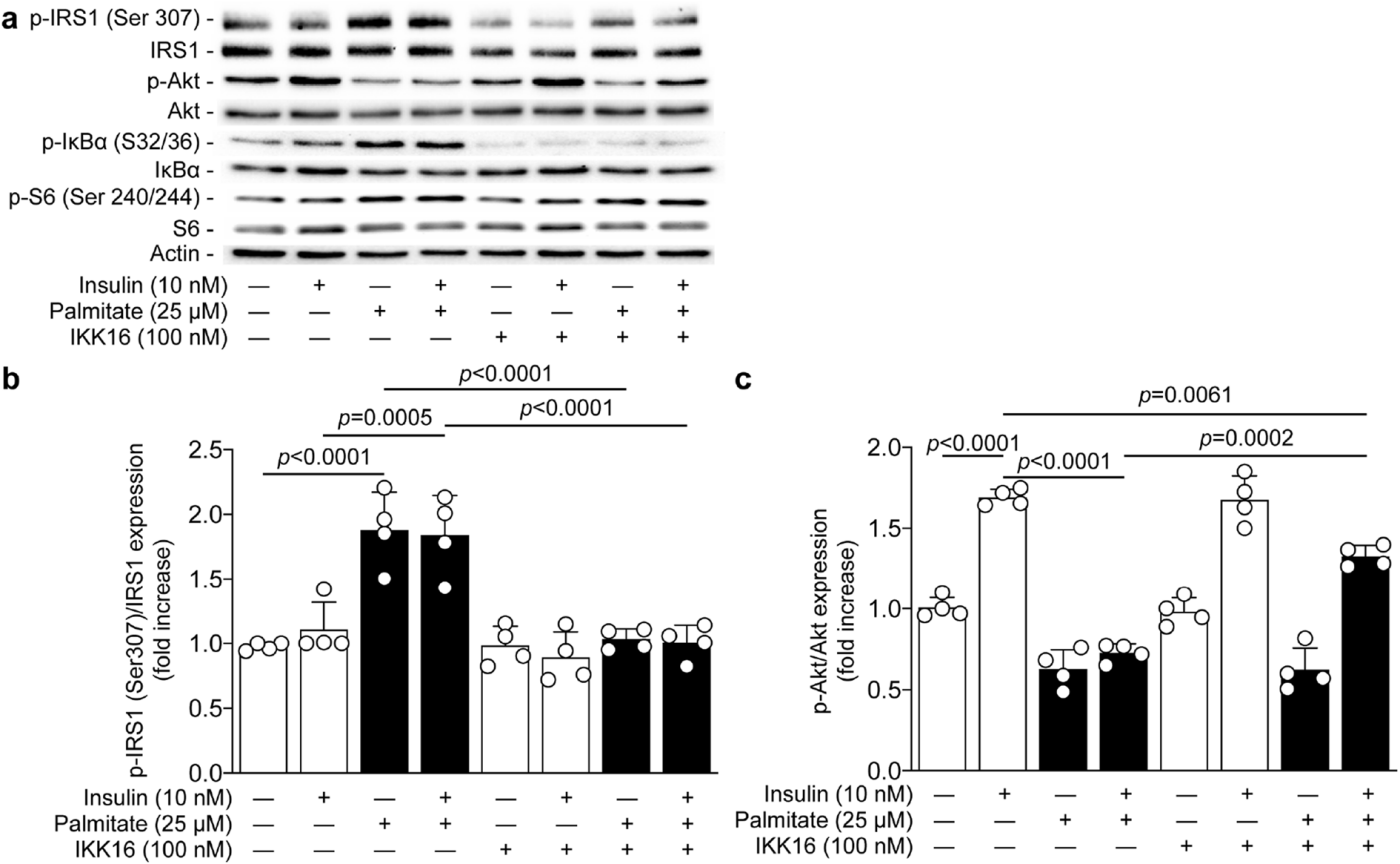
IKK inhibition prevented serine 307 phosphorylation of IRS1 and partially restored insulin-mediated Akt phosphorylation. Expression of phospho-IRS1 (ser307), IRS1, phospho-Akt, Akt, phospho-IκBα (ser32/36), IκBα, phospho-S6 (ser240/244), S6, and actin were detected by (**a**) immunoblot and (**b**, **c**) densitometry quantitation was measured from mouse podocytes treated or not with IKK16 (an IKK inhibitor) 30 min prior the exposure of palmitate for 24 h, and then stimulated with insulin for 5 min. Results are shown as mean ± SD of 4 independent experience.

To better correlate the activation of IKK to insulin resistance, we treated podocytes with the selective IKK inhibitor (IKK 16). Podocytes were treated with IKK 16 at 100 nM prior to exposure to palmitate and insulin stimulation. Our data showed that inhibition of IKK complex completely abolished the phosphorylation of IκBα on serine 32/36 in podocytes exposed to palmitate (Fig. 4a). Inhibition of IKK also totally prevented palmitate-induced phosphorylation of serine 307 of IRS1 (*p* < 0.0001; Fig. 4b), an effect that was associated with a partial restoration (59%) of insulin action on Akt activation compared to podocytes exposed to palmitate (*p* = 0.0001; Fig. 4c). It has been proposed that IKKβ may mediate pro-apoptotic function through the activation of S6 kinase. However, our data indicated that inhibition of IKK had no impact on palmitate-induced phosphorylation of S6 protein, a downstream of S6K1 (Fig. 4a).

### Ceramide synthesis inhibition blunted activation of IKKβ and re-established the insulin signaling pathway

**Figure 5:**
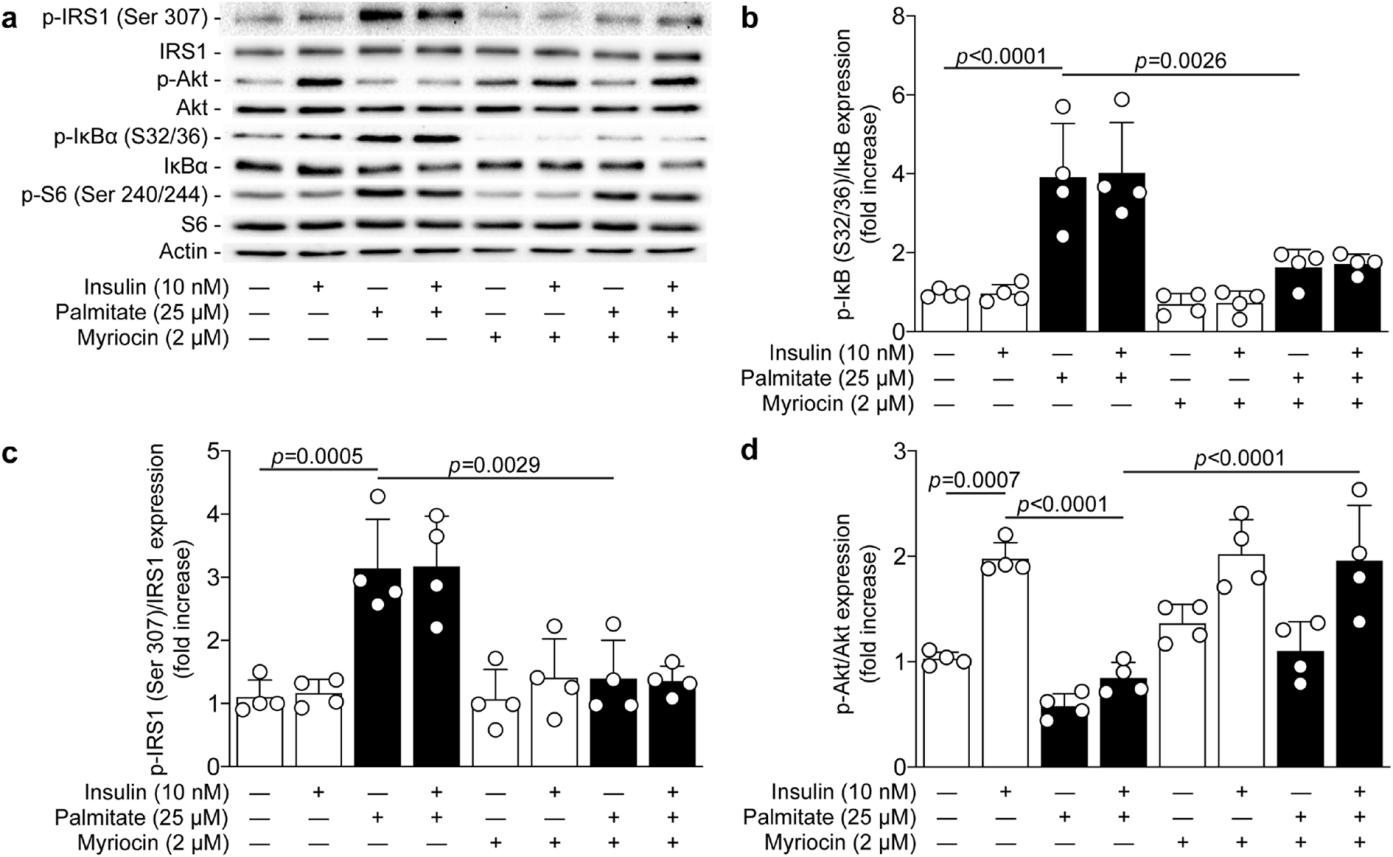
Palmitate-induced insulin resistance is blocked by ceramide synthesis inhibitor. Expression of phospho-IRS1 (ser307), IRS1, phospho-Akt, Akt, phospho-IκBα (ser32/36), IκBα, phospho-S6 (ser240/244), S6, and actin were detected by (**a**) immunoblot and (**b**, **c**, **d**) densitometry quantitation was measured from mouse podocytes treated or not with myriocin 30 min prior the exposure of palmitate for 24 h, and then stimulated with insulin for 5 min. Results are shown as mean ± SD of 4 independent experience.

Palmitate can be metabolized into ceramides, which is known to potentiate activation of the NF-κB pathway^28^. Moreover, inhibition of ceramide synthesis has been shown to prevent palmitate-induced inhibition of glucose uptake in podocytes^20^. In order to unravel the mechanism of action of palmitate-induced insulin inhibition in podocytes, cells were treated with myriocin, a ceramide synthesis inhibitor. While podocytes exposed to palmitate increased serine 32/36 phosphorylation of IκBα by 3.9-fold (*p* < 0.0001), inhibition of ceramide synthesis with myriocin significantly reduced IκBα phosphorylation by 78% (*p* = 0.0026), reaching almost similar levels to non-exposed podocytes (Fig. 5a,b). Moreover, myriocin treatment completely prevented palmitate-induced phosphorylation of serine 307 of IRS1 (*p* = 0.0029, Fig. 5c), thereby restoring insulin-induced Akt activation (*p* < 0.0001) in podocytes to a similar level of the untreated cells (Fig. 5d). These effects were independent of mTORC1 activation, since myriocin treatment did not prevent palmitate-induced S6 phosphorylation on serine 240/244 (Fig. 5a).

### Palmitate-induced inhibition of insulin actions is restored by blocking mTORC1 activation

**Figure 6:**
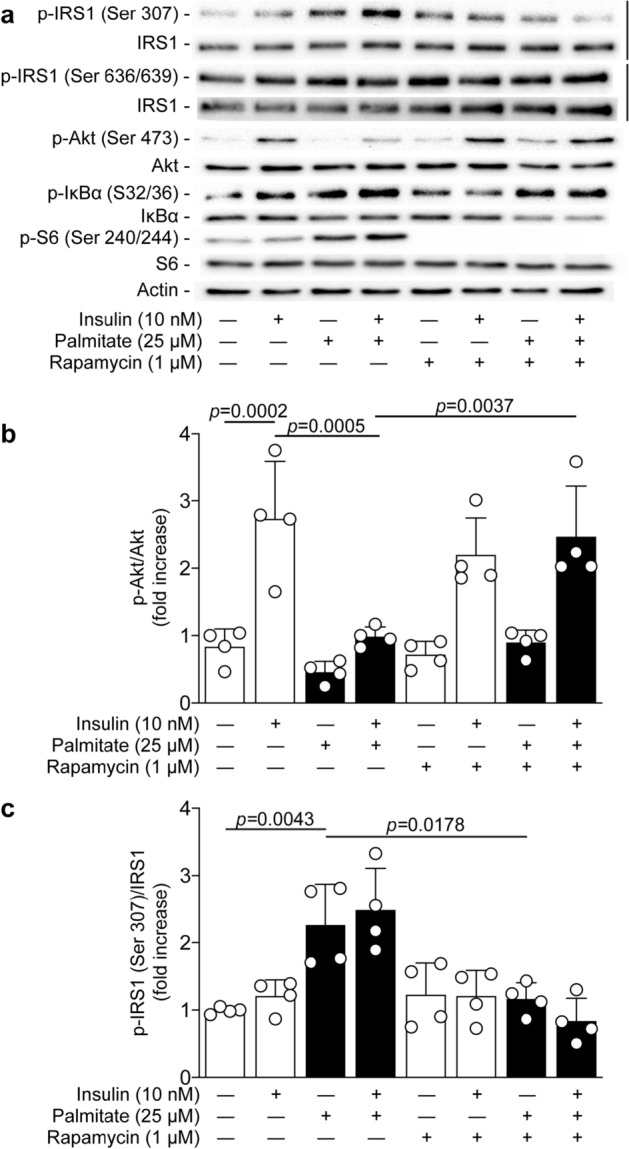
Inhibition of mTORC1 activation by rapamycin restored insulin actions in podocytes exposed to palmitate. Expression of phospho-IRS1 (ser307), IRS1, phospho-Akt, Akt, phospho-IκBα (ser32/36), IκBα, phospho-S6 (ser240/244), S6, and actin were detected by (**a**) immunoblot and (**b**, **c**) densitometry quantitation was measured from mouse podocytes treated or not with rapamycin 30 min prior the exposure of palmitate for 24 h, and then stimulated with insulin for 5 min. Results are shown as mean ± SD of 4 independent experience.

Activation of S6K1 by mTORC1 has been linked to insulin resistance through serine 307 phosphorylation of IRS1^29,30^. Palmitate exposure in podocytes caused a 2.1-fold increase in phosphorylation of the S6 ribosomal protein at serine 240/244 (*p* < 0.0001). Therefore, we investigated the effect of mTORC1 inhibition on the insulin signaling pathway. Treatment with rapamycin almost completely restored (90%) insulin-induced Akt phosphorylation in podocytes exposed to palmitate (*p* = 0.0037; Fig. 6a,b). In addition, rapamycin treatment prevented serine 307 phosphorylation of IRS1 induced by palmitate exposure by 87% (*p* = 0.0178; Fig. 6a,c) with no effect on serine 636/639 of IRS1. Interestingly, inhibition of mTORC1 (measured by the phosphorylation of serine 240/244 of S6) had no effect on IKK activation, since rapamycin treatment failed to prevent palmitate-induced serine 32/36 phosphorylation of IκBα (Fig. 6a).

## Discussion

**Figure 7:**
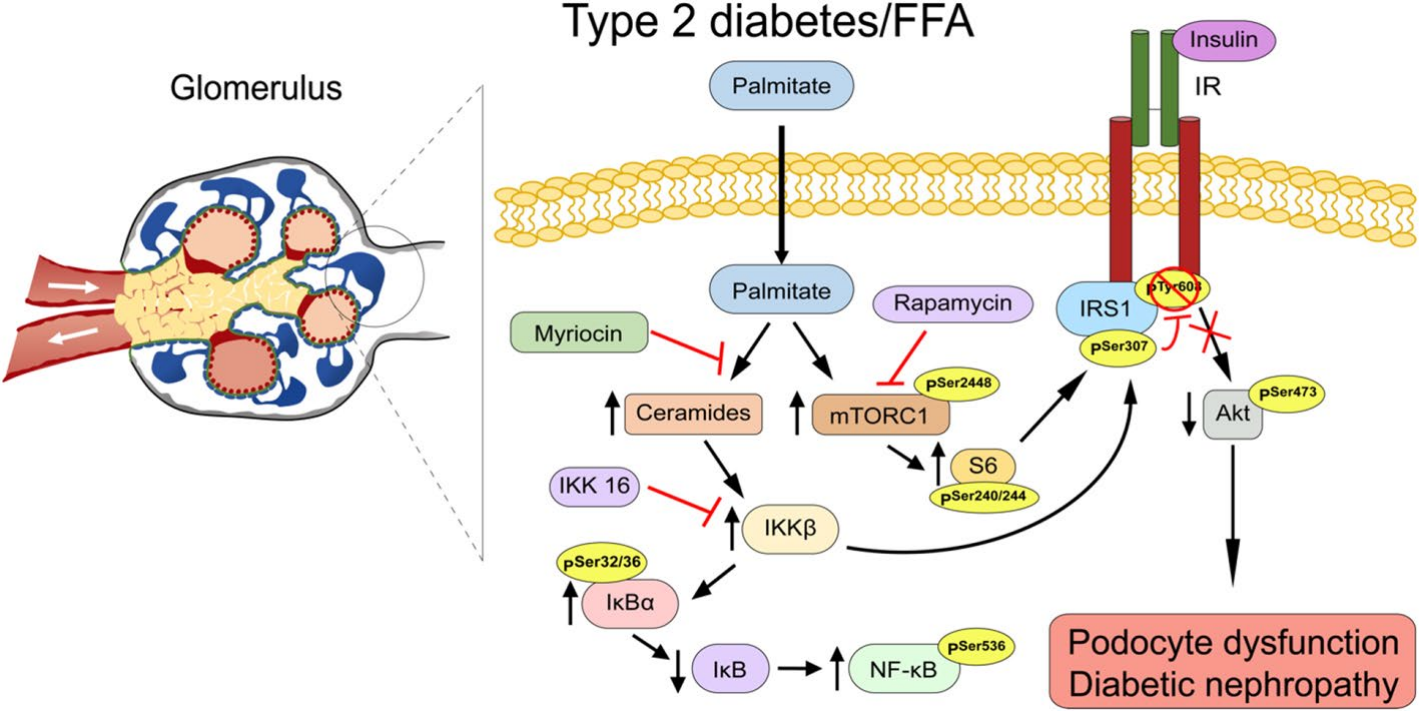
Schematic representation of the insulin signaling resistance caused by palmitate treatment in podocytes.

Although hyperglycemia has been shown to a play critical role in podocyte dysfunction, these epithelial cells are also vulnerable to injury from saturated FFA. A potential mechanism of FFA leading to podocyte dysfunction is through the inhibition of insulin actions. Podocytes are highly sensitive to insulin^31^. We and other groups have published that insulin unresponsiveness or absence of the insulin receptor is crucial for podocyte function and survival^17,32^. In this study, we provided novel insights into how FFA can cause insulin resistance in podocytes. Our data demonstrated that palmitate increased serine 307 phosphorylation of IRS1, which consequently reduced insulin stimulated Akt phosphorylation. We have uncovered that palmitate contributes to IRS1 inhibition through activation of IKK and mTORC1 but not PKC and JNK (Fig. 7). Importantly, these two pathways operate independently, since inhibition of each pathway with their respective selective inhibitor was able to restore insulin signaling.

Multiple studies in rodent models of obesity and type 2 diabetes revealed the central role of IRS1 serine phosphorylation in the development of insulin resistance. However, not all animal models of type 2 diabetes are suitable to study diabetic nephropathy. In our study, we used the leptin deficient mice as a proper model whereas the high fat diet mouse model did not develop renal pathology (data not shown). Nonetheless, efforts from various groups led to the identification of multiple serine kinases, in which the activity has been shown to be increased in a context of obesity and diabetes. These kinases, including proteins from the PKC family, JNK, IKK-β and S6 kinase are capable of directly interacting with IRS1^33^. Multiple evidence suggests that the IKK/NF-κB pathway plays a role in the induction and preservation of a chronic inflammatory state, which contributes to metabolic disorders such as obesity and type 2 diabetes^34,35^. NF-κB forms a complex with IκBα, an IκB family member, in the cytosol to prevent activated NF-κB from entering the nucleus to initiate DNA transcription. However, the activation of the IKK complex by various stimuli provokes the phosphorylation of IκBα molecules causing their degradation and the release of NF-κB. Previous studies have reported that high glucose levels and advanced glycation end-product exposure in podocytes caused reactive oxygen species production through NF-κB activation^36,37^. Our study is the first to demonstrate that palmitate is also able to phosphorylate IκBα and subsequently activate NF-κB in podocyte as well as in the renal cortex of type 2 diabetic mice compared to non-diabetic littermate controls. Interestingly, in response to an oxidative stress signal, IKKβ can provoke pro-apoptotic functions through the activation of p85 S6K1^38^. Thus, it would have been possible that IKK and mTORC1/S6K1 interact with each other and promote insulin resistance. However, our data suggest both pathways affect serine phosphorylation of IRS1 independently. Further studies will be required to evaluate if IKK signaling pathway activated by palmitate can regulate podocyte function through modulation of the mTOR/autophagy cascade.

Elevated levels of palmitate correlate with increased production of ceramides. Both endogenous and exogenous ceramides can contribute to the insulin resistance phenomenon. Previous studies reported that administration of myriocin blocked homocysteine- and high-fat diet-induced glomerular injury in the kidney^39,40^. In addition, palmitate exposure in cultured podocytes led to increased ceramide production^40^. Inhibition of ceramide synthesis using fumonosin B1 or myriocin partially prevented palmitate-induced inhibition of glucose uptake following insulin stimulation^41^. Therefore, we have investigated the impact of ceramide production on insulin signaling in podocytes. Our study demonstrated that the inhibition of ceramide synthesis with myriocin in podocytes exposed to palmitate prevented IRS1 serine phosphorylation and restored insulin signaling actions. Interestingly, previous studies revealed that ceramides can activate the IKK/NFκB pathway in alveolar epithelial cells and platelets^42,43^. Our results support the notion that increased ceramide production by palmitate metabolism leads to activation of IKKβ and contributes to insulin resistance through inhibition of IRS1 in podocytes.

Another potential mechanism of podocyte dysfunction in diabetic nephropathy is the regulation of mTOR activity. Very elegant animal studies have demonstrated the importance of regulating mTORC1 activity^44^. Evidence suggests that mTORC1 activation is elevated in podocytes of diabetic rodent and human kidneys. Inhibition of mTORC1 preserved podocyte function in DN^45^. Although inactivation of mTORC1 seems to be a promising avenue to treat diabetic kidney disease, reduction of basal mTORC1 activity in podocytes led to proteinuria and podocyte injury, suggesting that complete inhibition of mTORC1 activity is not a suitable strategy for DN treatment^46^. Nonetheless, hyperactivity of mTORC1 has been previously shown in podocytes from crescentic glomerular diseases^47^. Our data indicate that diabetes increased mTOR and S6 phosphorylation in renal glomeruli of *db/db* mice as compared to non-diabetic littermate controls. The elevated phosphorylation of S6 and serine 307 of IRS1 in podocytes exposed to palmitate were blunted by rapamycin and ceramide synthesis inhibitors, which restored insulin-mediated Akt phosphorylation. Interestingly, our data indicated that mTORC1/S6 activation mainly increased serine 307 phosphorylation, without affecting other known serine phosphorylation of IRS1 and Grb10, contrasting with previous observation in other insulin-sensitive cells^11,48^. Our results also corroborate previous studies showing that palmitate regulated podocyte apoptosis through mTORC1 lysosomal localization^24^. Interestingly, Kumar and collaborators previously showed that short treatment of rapamycin prevented mTORC1-induced insulin resistance in human podocytes, an effect that was associated with decreased expression of IκBα and phosphorylation NF-κB^23^. This is in contrast to our study that did not show inhibition of IκBα phosphorylation with rapamycin. Potential explanations for this discrepancy are the use of different podocyte cell lines and the exposure time to rapamycin (short versus long exposure).

In conclusion, our results showed that inhibition of insulin signaling by palmitate in podocytes is associated with serine 307 phosphorylation of IRS1. Moreover, we identified two independent mechanisms activated by palmitate that resulted in phosphorylation of IRS1 on serine 307. Overall, our results suggest that IRS1 phosphorylation on serine 307 could be a target of interest to prevent podocyte dysfunction in a context of obesity and diabetes.

## Research design and methods

### Reagents and antibodies

Primary antibodies for immunoblotting and immunochemistry were obtained from commercial sources, including actin (horseradish peroxidase conjugated [HRP]; I-19), GAPDH-HRP (V18), phospho NF-κB (Ser 536) (101,752), NF-κB (F-6), phospho-JNK (G-7), JNK (D-2), PKCα (C-20), PKCβ (C-18), PKCε (C-15) and PKCζ (C20), from Santa Cruz Biotechnology (Santa Cruz, CA, USA); phospho-Akt (Ser473) (D9E), Akt (9272), phospho-IκB (S32/36) (5A5), IκBα (9242), phospho-mTOR (Ser2448) (D9C2), mTOR (7C10), phospho-S6 ribosomal protein (Ser240/244) (2215), S6 ribosomal protein (5G10), phospho-IRS1 (Ser636/639) (2388) and phospho-Grb10 (Ser476) (D4E6) and PKCδ (2058) from Cell Signaling (Beverly, MA); phospho-IRS1 (Ser307) (05-1087); phospho-IRS1 (Tyr608) (09-432); IRS1 (05-1085) from Millipore-Sigma (Oakville, ON, Canada), Grb10 (Abcam; ab154029), collagen type IV (Novus Biological, Littleton, CO). All other reagents employed, including RPMI-1640, EDTA, leupeptin, phenylmethyl-sulfonyl fluoride, aprotinin, d-glucose, d-mannitol, FITC-inulin and Na3VO4 were purchased from Millipore-Sigma, fetal bovine serum (FBS) was purchased from Wisent bioproducts (Saint-Jean-Baptiste, QC, Canada) and penicillin–streptomycin was obtained from Invitrogen.

### Animals and experimental design

C57BLKS/J (*db/dm*) and obese diabetic male C57BKS-Lepr^−^/^−^ (*db/db*) mice were purchased from The Jackson Laboratory and bred in our animal facility. All experiments were done on mice at 25 weeks of age. All experiments were conducted in accordance with the Canadian Council of Animal Care and Institutional Guidelines and were approved by the Animal Care and Use Committees of the University of Sherbrooke according to National Institutes of Health guidelines.

### Blood glucose, urinary albumin/creatinine ratio and glomerular filtration rate measurements

Blood glucose and body weights were measured by a Glucometer (Contour; Bayer, Pointe-Claire, QC, Canada) at 24 h prior sacrifice (Supplementary Table 1). Urine was collected at day of sacrifice to measure albumin and creatinine levels. Urinary albumin levels were measured using an indirect competitive enzyme-linked immunosorbent assay (ELISA) according to the manufacturer’s instructions (Albuwell M; Exocell; Philadelphia, PA). Creatinine levels were measured using alkaline picrate coloration based on Jaffe reaction per manufacturer’s instruction (The Creatinine Companion; Exocell). Glomerular filtration rate (GFR) was evaluated using FITC-inulin clearance as we previously described^49^.

### Tissue preparation

Right mouse kidneys were harvested for pathological examination, and sections were fixed in 4% paraformaldehyde (Millipore-Sigma) and then transferred to 70% ethanol for immunohistochemistry. The tissue was embedded in paraffin, and 4 µm sections were stained with hematoxylin & eosin and periodic acid-Schiff stain (Millipore-Sigma).

### Immunohistochemistry, mesangium expansion and glomerular hypertrophy

Immunochemistry of kidney sections was performed using the ABC kit from Vector according to manufacturer protocol. Coloration was obtained by incubating sections in DAB solution (Vector Laboratories Inc.; Burlington, ON, Canada). Nuclei were counterstained using Gill’s formula hematoxylin (Vector Laboratories Inc.). Mesangial matrix expansion and glomerular hypertrophy were measured as we previously described^49^.

### Systemic injection of insulin

Insulin signaling pathway was evaluated by injecting 5 mU/g I.V. in *db/dm* and *db/db* mice. The right kidney was removed prior to insulin injection and served as an internal control (non-stimulated). Fifteen minutes after the insulin injection, the left kidney was removed for protein extraction.

### Isolation of the glomeruli

Renal cortex of one kidney (1 per mouse) were minced and all the tissue was passed through a 200, 150, 70 and 40 µm sieve. The glomeruli remained at the top of 70 µm sieve. The glomeruli fraction was collected with PBS and centrifuge for 10 min at 500 g. The glomeruli samples (1 kidney per N, total of N = 4 per group) were then used for immunoblot analyses.

### Immunoblot analyses

Protein lysate (10–50 µg) was separated by SDS-PAGE and then transferred to a PVDF membrane, which was blocked with 5% skim milk. Proteins were identified by chemiluminescence using Forte solution (Millipore).

### Palmitate preparation

Palmitate was prepared as previously described^50^. Briefly, palmitic acid was diluted in dH_2_O 0.1 N NaOH then heated to 70 °C to dissolve. In parallel, 10% BSA in dH_2_O was heated to 55 °C. When dissolved, palmitate was quickly added to heated 10% BSA solution to create a 1:6 palmitate: BSA solution. Stock palmitate: BSA solution was sterile filtered (0.22 microns) before usage. Palmitate concentration of stock solution was measured using HR series NEFA-HR(2) (Wako Diagnostics USA, Moutain View, CA).

### Cell culture

A well-characterized mouse podocyte cell line was used and cultured as previously described^32^. After differentiation of podocytes, medium was changed to RPMI 0.1% FBS containing 5.6 mmol/L of glucose for 48 h, with or without 25 μmol/L of palmitate during the last 24 h before harvesting proteins. Rapamycin (1 μmol/L), IKK16 (100 nmol/L) or Myriocin (2 μmol/L) was added during the last 24 h.

### Statistical analyses

In vitro and in vivo data are shown as mean ± SD for each group. Statistical analysis was performed by unpaired *t* test or by one-way analysis of variance (ANOVA) followed by Tukey’s test correction for multiple comparisons. Data in each group were checked for normal distribution using D’Agostino and Pearson normality test based on alpha = 0.05. All results were considered statistically significant at *p* < 0.05.

## Supplementary Information

Supplementary Table.
