# Supplementary material for: Saturated fatty acids induce insulin resistance in podocytes through inhibition of IRS1 via activation of both IKKβ and mTORC1
Source: Sci Rep. 2020 Dec 10;10:21628. doi: 10.1038/s41598-020-78376-1 (PMC7728775; doi:10.1038/s41598-020-78376-1)
Supplement: Supplementary file 1 — Supplementary Table. [file 41598_2020_78376_MOESM1_ESM.docx]

Saturated fatty acids induce insulin resistance in podocytes through inhibition of IRS1 via activation of both IKKβ and mTORC1

Benoit Denhez^1^, Marina Rousseau^1^, Crysta Spino^1^, David-Alexandre Dancosst^1^, Marie-Ève Dumas^1^, Andréanne Guay^1^, Farah Lizotte^1^, Pedro Geraldes^1,2△^

Supplemental material

**Supplementary Table 1. Body weight and blood glucose levels.**

|  | *db/dm* | *db/db* |
| --- | --- | --- |
| **Weight (g)** | 32.27 ± 4.19 | 49.38 ± 8.38 |
| **Glucose (mg/dL)** | 133.8 ± 44.8 | 541.1 ± 63.0 |
